# Outcomes 1 year after a first episode of psychosis in migrants to the Republic of Ireland

**DOI:** 10.1177/00207640231174360

**Published:** 2023-05-21

**Authors:** Brian O’Donoghue, Eric Roche, John Lyne, Laoise Renwick, Mary Clarke

**Affiliations:** 1 Department of Psychiatry, University College Dublin, Ireland; 2 Department of Psychiatry, Royal College of Surgeons in Ireland, Dublin, Ireland; 3 Centre for Youth Mental Health, University of Melbourne, Parkville VIC, Australia; 4 Cluain Mhuire Mental Health Services, Newtownpark Avenue, Blackrock, Co. Dublin, Ireland; 5 Wicklow Mental Health Services, Newcastle Hospital, Greystones, Co. Wicklow, Ireland; 6 Division of Nursing, Midwifery and Social Work, Faculty of Biology, Medicine and Health, University of Manchester, UK; 7 DETECT Early Intervention for Psychosis Service, Blackrock, Co. Dublin, Ireland

**Keywords:** Psychosis, schizophrenia, outcomes, migrants, remission

## Abstract

**Background:**

Migration is a robust risk factor for developing a psychotic disorder, yet there is a paucity of research on the outcomes of migrants who develop a psychotic disorder. Identifying sub-groups within FEP cohorts who have a poorer outcome, could assist in the development and delivery of more targeted interventions.

**Aims:**

There is a paucity of research on the outcomes of migrants who develop a psychotic disorder. This study aimed to evaluate a broad range of outcomes for those with a FEP who migrated to the Republic of Ireland, including: (i) symptomatic; (ii) functional; (iii) hospitalisation and (iv) engagement with psychosocial services.

**Methods:**

All individuals with a FEP aged 18 to 65 who presented between 01.02.2006 and 01.07.2014 were included. Structured and validated instruments were used to measure positive, negative, depressive symptoms and insight.

**Results:**

Of the 573 individuals with a FEP, 22.3% were first-generation migrants and 63.4% (*n* = 363) were followed up at 1 year. At this time, 72.4% of migrants were in remission of positive psychotic symptoms compared to 78.5% of the Irish born (*OR* = 0.84, 95% CI [0.50–1.41], *p* = .51). In relation to negative symptoms, 60.5% of migrants were in remission compared to 67.2% of the Irish born (*OR* = 0.75, 95% CI [0.44–1.27], *p* = .283). There was no difference in the severity of positive, negative or depressive symptoms between groups and there was a trend for the Irish born to have better insight (*p* = .056). The functional outcomes were similar across groups. One third of migrants were admitted to hospital compared to 28.7% of the Irish born (*OR* = 1.24, 95% CI [0.73–2.13], *p* = .426). Just over half of both groups attended CBT and 46.2% of caregivers for migrants attended the psychoeducation programme, compared to 39.7% for the Irish born (*OR* = 1.30, 95% CI [0.79–2.16], *p* = .306).

**Conclusions:**

These findings demonstrate that migrants have broadly similar outcomes to the native-born populations, however there is still considerable scope for the outcomes for all individuals affected by psychotic disorders to be improved.

## Introduction

Migration is one of the most robust and replicated risk factors for the development of a psychotic disorder (Selten et al., 2020), yet there is a relative paucity of research on the outcomes of migrants who develop a first episode of psychosis (FEP). Migrants with a FEP are more likely to have a preponderance of factors associated with a poorer prognosis, such as a prolonged duration of untreated psychosis (Nerhus et al., 2015), more severe psychotic symptoms at presentation (Waxmann et al., 2022) and higher disengagement rates (Ouellet-Plamondon et al., 2015). In addition, they are more likely to have negative experiences during their pathway to care, such as higher rates of attendance at emergency departments and involuntary admission (Waxmann et al., 2022). Therefore, it would be reasonable to hypothesise that migrants would have poorer outcomes compared to the native-born populations following the onset of a psychotic disorder. Yet, a systematic review of the limited literature found that migrants with a FEP can have better outcomes in some domains and more negative outcomes in other domains (Maguire et al., 2020).

In relation to the better outcomes, migrants have been found to have either a similar level of severity of positive psychotic symptoms at follow-up (Abdel-Baki et al., 2018) or even higher rates of remission (Zandi et al., 2011). Furthermore, risk of death by suicide was found to be lower in migrants following the onset of a FEP (Termorshuizen et al., 2012). However, migrants have been found to have higher rates of disengagement (Ouellet-Plamondon et al., 2015) and involuntary admission in the period following the initial presentation (Rodrigues et al., 2019). Therefore, within the limited literature, there are conflicting and not yet understood clinical findings in relation to the outcome of migrants with a FEP. The aforementioned systematic review focused exclusively on the outcomes of individuals with a FEP who were migrants and thereby did not include studies that examined the outcomes of ethnic minorities, unless migrant status was stated. Specific ethnic minorities have been found to have poorer outcomes, for example, after a FEP, Black Caribbean individuals were less likely to achieve symptomatic recovery, be employed or be in a relationship (Morgan et al., 2017).

While it is likely that different outcomes are inter-related, individual studies have tended to examine specific outcomes in isolation. For example, individuals who disengage from services and subsequently relapse, may experience severe symptoms, making an involuntary admission more likely. The DETECT Early Intervention for Psychosis Service (EIS) was established in 2006 and provides assessment and treatment to individuals aged 18 to 65 presenting with a FEP in South Dublin and parts of county Wicklow. The establishment of this service also coincided with a period of net migration into Ireland since 1996 (Gilmartin, 2012). Nearly one quarter (22.3%) of the individuals identified as experiencing a FEP by this service were first-generation migrants, coming from a wide range of countries (O’Donoghue et al., 2021),

Therefore, this study aimed to determine the outcomes for these individuals who developed a FEP following migration to the Republic of Ireland. Outcomes were assessed across a broad range of domains, including: (i) symptomatic outcomes, specifically proportion who achieve remission and severity of positive and negative symptoms of psychosis, depressive symptoms and insight; (ii) functional outcomes; (iii) hospitalisation, specifically admission rates, including involuntary admissions and total bed days (iv) Engagement with psychosocial services, specifically cognitive behavioural therapy (CBT) and whether their caregivers attended a psychoeducation programme.

## Methodology

### Setting and participants

This study was conducted at the Dublin and East Treatment and Early Care Team (DETECT) Early Intervention for psychosis service, which encompasses three mental health services in South Dublin and Co Wicklow and covers a total population of ~377,000 people. All individuals with a FEP aged 18 to 65 years who presented between February 2006 and July 2014 were included.

### Inclusion and exclusion criteria

All individuals with a psychotic disorder, according to DSM-IV criteria, except those with a diagnosis of psychosis due to a general medical condition, were included. A first episode of psychosis was defined as an incident case of psychosis when the individual had not previously experienced a psychotic episode and, before referral, had not previously prescribed antipsychotic medication for more than 30 days.

### Definitions

Individuals were classified as first-generation migrants if they were born in a country other than the Republic of Ireland. We did not have sufficient information to determine whether an individual was a second-generation migrant, and therefore, any potential second-generation migrants were included in the ‘born in the Republic of Ireland’ group. However, the Republic of Ireland started to receive migrants at a much greater level in the last 1990s and early 2000s, therefore the population was relatively homogeneous prior to this, with the vast majority of the population in the Republic of Ireland identifying as White Irish in the 1996 and 2001 census respectively (Central Statistics Office, 1996, 2001). As psychotic disorders have their peak onset in late adolescence and early adulthood and this study included individuals who presented with a FEP between 2006 and 2014, it would be very unlikely that there would be a significant number of second generation migrants in the ‘born in the Republic of Ireland’ group.

The definition of remission was taken from the consensus definition from an expert panel led by Andreasen et al. (2005), that has been validated in first episode cohorts (Cassidy et al., 2010). The criteria for remission of positive symptoms were a score of 2 or less on the global scores for each positive symptoms domain, specifically hallucinations, delusions, bizarre behaviour and thought disorder. Similarly, the criteria for remission of negative symptoms were a score of 2 or less on the global scores for affective flattening, alogia, avolition and anhedonia. However, the duration component of the criteria for remission was not applied, as we could not determine how long an individual was in remission, as symptom severity was only measured at baseline and at 1 year follow-up.

### Instruments

The Structured Clinical Interview for Diagnostic and Statistical Manual of Mental Disorders IV (SCID) was used to determine the psychotic disorder diagnosis and the presence of any concurrent substance use disorder (13). The SCID also included the Global Assessment of Functioning (GAF) which has a range of 0 to 100, with higher scores indicating higher functioning (First et al., 2002). Positive psychotic symptoms were measured using the Scale for the Assessment of Positive Symptoms (SAPS), which examines four main domains (delusions, hallucinations, bizarre behaviour and formal thought disorder). Each domain is scored from 0 (absent) to 5 (severe) and the total score for the SAPS ranges from 0 to 20 (Andreasen, 1984b). Negative symptoms were measured using the Scale of the Assessment of Negative Symptoms (SANS), which measures five domains (affective flattening, alogia, avolition-apathy, anhedonia-asociality and attention) and each domain is scored from 0 (absent) to 5 (severe) for a total score of 0 to 25 (Andreasen, 1984a). Depression was measured using the Calgary Depression Scale for Schizophrenia (CDSS), which contains nine items, each scored from 0 (absent) to 3 (severe) for a total score of 0 to 27 (32). Insight was measured using the Birchwood Insight Scale, which is a self-reported scale that is scored from 0 to 12 with higher scores indicating greater insight (Birchwood et al., 1994). Interventions provided by the EIS were included a 12 session Cognitive Behavioural Therapy (CBT) programme and a six session Family Psychoeducation Programme. Both of these interventions were delivered in group format due to resource constraints.

### Ethical approval

This study received ethical approval from the St John of God Hospitaller services ethics committee (Application ID 665). Individuals provided informed consent to participate in the 1-year follow-up assessment.

### Statistical analysis

Data were determined to be parametric or non-parametric by examining the skewness of the data. If the ratio of skewness to the standard error of skewness was ⩾1.96, then the data was considered to be non-parametric and the non-parametric equivalent statistical test was utilised. *T*-tests were conducted to determine differences between parametric continuous variables and Mann-Whitney *U* tests were used to determine if there were differences in medians. Chi-square tests (χ^2^) were used to determine whether there was a difference in the observed outcome in categorical variables and if there were less than five cases in any group, Fishers exact test was used to determine statistical significance. Binary logistic regression was used to determine the odds ratios and 95% confidence intervals for the predictor variables being associated with the outcome variable. Statistical analysis was conducted with SPSS v28.0. The data are not publically available, however queries can be directed to the corresponding author.

## Results

### Description of participants and comparison of those followed up and those not followed up

A total of 573 individuals presented with a FEP during the study period and of these, 63.4% (*n* = 363) had a follow-up assessment at 1 year after presentation. Comparisons of the demographic and clinical characteristics of the individuals who had a follow-up assessment compared to those who were not followed up are presented in Table 1. Individuals who were treated as an outpatient at the time of presentation as opposed to being admitted were more likely to have a follow-up assessment (68% vs. 60%, *p* = .034). In addition, there was a non-significant trend for individuals who were lost to follow-up having less insight (median 7.0 vs. 8.0, *p* = .066) and less severe positive symptoms (median 7.0 vs. 8.0, *p* = .084) at presentation. A comparison of the demographic and clinical characteristics between the Irish born cohort and migrants at the time of presentation with a FEP were described in a previous publication (O’Donoghue et al., 2021). Of note, there were no differences at baseline between these two groups, except that migrants from Africa presented with lower levels of insight.

**Table 1.** Demographic and clinical characteristics of the total cohort and a comparison of those followed up at 1 year and those lost to follow-up.

	Total cohort		Follow-up completed		Not followed-up			
Sex	%	*n*	%	*n*	%	*n*	χ^2^, *df*	*p*
Male	55.8	320	54.5	198	58.1	122	.68, 1	.410
Female	44.2	253	45.5	165	41.9	88		
Age	Median	I.Q.R.	Median	I.Q.R.	Median	I.Q.R.	*Z*	
Age at presentation	32.0	24.0–43.0	32.0	25.0–43.0	31.0	24.0–41.3	−1.01	.313
Age at onset	29.8	21.8–39.3	29.8	21.5–39.3	29.4	22.2–39.4	−0.35	.727
Marital status	%	*n*	%	*n*	%	*n*	χ^2^, *df*	
Single	68.5	392	65.0	236	74.6	156	5.74, 2	.057
Married/de factor	22.9	131	25.6	93	18.2	38		
Separated/Divorced/Widowed	8.6	49	9.4	34	7.2	15		
Employment status								
Employed	36.6	210	36.6	133	36.7	77	.001, 1	.995
Unemployed	63.4	363	63.4	230	63.3	133		
Migrant status								
First generation migrant	22.3	128	21.8	79	23.3	49	.19, 1	.664
Born in the Republic of Ireland	77.7	445	78.2	284	76.7	161		
Place of birth								
Ireland	77.7	445	78.2	284	76.7	161	1.41, 4	.843
Rest of Europe	13.4	77	13.2	48	13.8	29		
Asia	3.7	21	3.9	14	3.3	7		
Africa	2.8	16	8	2.2	8	3.8		
Americas	2.4	14	9	2.5	5	2.4		
Inpatient admission at presentation								
Inpatient at presentation	62.0	355	58.7	213	67.6	142	4.51, 1	.034
Outpatient at presentation	38.0	218	41.3	150	32.4	68		
Diagnosis*								
*Non-affective psychotic disorders*							5.54, 1	.019
Schizophreniform disorder	11.9	68	10.5	38	14.3	30		
Schizophrenia	28.3	162	29.8	108	25.7	54		
Delusional disorder	11.7	67	12.1	44	11.0	23		
Substance-induced psychotic disorders	14.3	82	13.2	48	16.2	34		
psychotic disorder	7.9	45	6.1	22	11.0	23		
Psychosis NOS	3.8	22	3.3	12	4.8	10		
*Affective psychotic disorders*								
Schizoaffective disorder	1.6	9	2.5	9	0	0		
Depression with psychosis	9.8	56	9.9	36	9.5	20		
Bipolar affective disorder	10.8	62	12.7	46	7.6	16		
Concurrent diagnoses								
Substance abuse or dependence present	31.2	179	30.3	110	32.9	69	.40, 1	.525
Alcohol abuse or dependence present	17.5	100	18.5	67	15.7	33	.70, 1	.405
Functioning	Median	I.Q.R.	Median	I.Q.R.	Median	I.Q.R.	*Z*	*p*
GAF total	30.0	25–40	30.0	25–40	35.0	25–40	−1.62	.105
Symptom severity								
Positive symptoms, Global SAPS score	8.0	5.0–10.0	8.0	5.0–10.0	7.0	4.0–9.0	−1.729	.084
Negative symptoms, global SANS score	4.0	1.0–8.0	4.0	1.0–8.0	4.0	0–8.0	−0.888	.375
Depressive symptoms, CDSS score	2.0	0–6.0	2.0	0–7.0	2.0	0–6.0	−0.356	.722
Insight	7.5	5.0–9.0	8.0	5.5–9.5	7.0	5.0–9.0	−1.837	.066

* The comparison for diagnosis is for non-affective compared to affective psychotic disorders.

### Symptomatic and functional outcomes at 1 year

A total of 72.4% (*n* = 55) of migrants were in remission of positive psychotic symptoms at 1 year follow-up compared to 78.5% (*n* = 212) of the Irish born cohort (*OR* = 0.84, 95% CI [0.50–1.41], *p* = .51). In relation to negative symptoms, 60.5% (*n* = 46) of migrants were in remission at 1 year follow-up compared to 67.2% (*n* = 182) of the Irish born cohort (*OR* = 0.75, 95% CI [0.44–1.27], *p* = .283). There were no differences in the severity of positive, negative symptoms or depressive symptoms at 1 year follow-up between the two groups (Table 2). There was a trend for Irish born individuals to have a higher median level of insight compared to migrants with a FEP (9.0 vs. 8.0, *Z* = −1.91, *p* = .056). At 1 year follow-up, there was no difference in the mean GAF scores between the two groups, which were between 60 and 64, indicating moderate difficulties in functioning. There was a similar proportion of individuals in employment at 1 year follow-up (36.6% vs. 38.7%, *OR* = 0.92, 95% CI [0.55–1.54], *p* = .744). All of the results were consistent within the sub-groups of migrants, according to the continental region of birth, except that the lower level of insight in migrants from Africa was statistically significant (median = 7.0, *Z* = −2.3, *p* = .021) (Table 3).

**Table 2.** One-year outcomes following a first episode of psychosis according to migrant status.

Outcome variable		Irish born cohort		Migrant population			*p*
Symptomatic outcomes	*N*	%	*N*	%	*N*	*OR* [95% CI]	
Remission of positive symptoms	346	78.5	212	72.4	55	0.84 [0.50–1.41]	.510
Remission of negative symptoms	347	67.2	182	60.5	46	0.75 [0.44–1.27]	.283
		Median	I.Q.R.	Median	I.Q.R.	*Z*	
Positive symptoms, SAPS Global score,	346	0	0–2.0	0	0–3.0	−1.50	.134
Negative symptoms, SANS Global score	345	2.0	0–7.0	3.0	0–8.25	−0.85	.398
Depression, CDSS total score	350	1.0	0–4.0	1.0	0–3.0	−0.10	.922
Insight, Birchwood total score	286	9.0	6.5–11.0	8.0	5.5–10.0	−1.91	.056
Functional outcomes		Median	I.Q.R.	Median	I.Q.R.	*Z*	
Global Assessment of functioning, GAF	356	65.0	49.0–81.5	62.0	45.0–75.0	−1.05	.294
		%	*N*	%	*N*	*OR* [95% CI]	
In competitive employment	363	38.7	110	36.7	29	0.92 [0.55–1.54]	.744
Hospitalisation		%	*N*	%	*N*	*OR* [95% CI]	
Admission to hospital, excluding at presentation	357	28.7	80	33.3	26	1.24 [0.73–2.13]	.426
Involuntary admission during first year	276	28.3	60	33.3	21	1.28 [0.70–2.33]	.430
		Median	I.Q.R.	Median	I.Q.R.	*Z*	
Median number of days in hospital in first year	311	24.0	0–53.0	21.0	0–52.0	−0.40	.692
Engagement with psychosocial interventions		%	*N*	%	*N*		
Attended Cognitive behavioural therapy	360	51.8	146	52.6	41	1.03 [0.63–1.71]	.902
Caregiver attended psychoeducation course	360	39.7	112	46.2	36	1.30 [0.79–2.16]	.307

**Table 3.** One-year outcomes following a first episode of psychosis according to migrant status classified at the continental level.

	Irish born	Rest of Europe		Asia		Africa		Americas	
Symptomatic outcomes	% (*N*)	% (*N*)	*OR* [95% CI]	% (*N*)	*OR* [95% CI]	% (*N*)	*OR* [95% CI]	% (*N*)	*OR* [95% CI]
Remission positive symptoms	78.5 (212)	70.8 (34)	0.81 [0.4–1.5]	64.3 (9)	0.88 [0.3–2.7]	62.5 (5)	0.49 [0.1–1.2]	77.8 (7)	1.70 [0.4–8.4]
Remission negative symptoms	67.2 (182)	64.6 (31)	0.81 [0.4–1.5]	50.0 (7)	0.49 [0.17–1.44]	62.5 (5)	0.49 [0.12–2.00]	88.9 (8)	1.71 [0.3–8.4]
	Median (I.Q.R.)	Median (I.Q.R.)	*Z*	Median (I.Q.R.)	*Z*	Median (I.Q.R.)	*Z*	Median (I.Q.R.)	*Z*
Positive symptoms, SAPS	0 (0–2)	0 (0–3.0)	−0.70	1.0 (0–6.25)	−1.44	1.5 (0.25–4)	−1.61	0 (0–4.0)	−0.09
Negative symptoms, SANS	2.0 (0–7)	2.0 (0–9.0)	−0.55	5.0 (0–8.25)	−0.94	4.5 (2.3–10.8)	−1.23	1.0 (0–2.75)	−1.01
Depression, CDSS	1.0 (0–4)	0 (0, 0)	−1.89	0 (0–1.0)	−0.16	1.0 (0.25–1.0)	−1.86	0 (0–0)	−1.12
Insight, Birchwood	9.0 (6.5–11)	8.0 (5.6–10.4)	−1.11	7.0 (4.4–9.75)	−1.69	7.0 (2.5–8.0)	−2.30*	9.5 (8.0–11.5)	−0.68
Functional outcomes	Median (I.Q.R.)	Median (I.Q.R.)	*Z*	Median (I.Q.R.)	*Z*	Median (I.Q.R.)	*Z*	Median (I.Q.R.)	*Z*
GAF	65.0 (49–81.5)	62.5 (45–73.8)	−0.84	61.0 (40.3–72)	−1.03	48 (41.3–68.3)	−1.51	75.0 (57–89.5)	−1.31
	% (*N*)	% (*N*)	*OR* [95% CI]	% (*N*)	*OR* [95% CI]	% (*N*)	*OR* [95% CI]	% (*N*)	*OR* [95% CI]
In competitive employment	38.7 (110)	33.3 (16)	0.79 [0.42–1.51]	42.9 (6)	1.20 [0.40–3.51]	12.5 (1)	0.23 [0.03–1.86]	66.7 (6)	3.16 [0.8–12.9]
Hospitalisation	% (*N*)	% (*N*)	*OR* [95% CI]	% (*N*)	*OR* [95% CI]	% (*N*)	*OR* [95% CI]	% (*N*)	*OR* [95% CI]
Admission to hospital	28.7 (80)	35.4 (17)	1.36 [0.72–2.60]	23.1 (3)	0.75 [0.20–2.78]	25.0 (2)	0.83 [0.16–4.20]	44.4 (4)	1.99 [0.5–7.6]
Invol adm during first year	28.3 (60)	30.6 (11)	1.12 [0.52–2.42]	54.5 (6)	3.06 [0.90–10.4]	28.6 (2)	1.02 [0.19–5.40]	22.2 (2)	0.73 [0.15–3.6]
	Median (I.Q.R.)	Median (I.Q.R.)	*Z*	Median (I.Q.R.)	*Z*	Median (I.Q.R.)	*Z*	Median (I.Q.R.)	*Z*
Median hospital days	24.0 (0–53)	20.0 (0, 53.5)	−0.57	14.0 (10–56)	−0.08	1.0 (0, 47)	−0.74	41.0 (7–58)	−0.69
Psychosocial interventions	% (*N*)	% (*N*)	*OR* [95% CI]	% (*N*)	*OR* [95% CI]	% (*N*)	*OR* [95% CI]	% (*N*)	*OR* [95% CI]
Attended CBT	51.8 (146)	48.9 (23)	0.89 [0.48–1.66]	57.1 (8)	1.24 [0.42–3.67]	50.0 (4)	0.93 [0.23–3.80]	66.7 (6)	1.86 [0.46–7.6]
Caregiver attended psychoeducation course	39.7 (112)	40.4 (19)	1.03 [0.55–1.93]	57.1 (8)	2.02 [0.68–6.0]	62.5 (5)	2.53 [0.59–10.8]	44.4 (4)	1.21 [0.32–4.6]

* *p* ⩽ .05. **p ⩽ .001.

### Hospitalisation

Excluding admissions at the time of presentation, one-third (33.3%, *n* = 26) of migrants were admitted to hospital during the first year after a FEP compared to 28.7% (*n* = 80) of the Irish born cohort (*OR* = 1.24, 95% CI [0.73–2.13], *p* = .426). There was missing data in relation to the legal status of admission for 22.7%, however there was no difference in the rates of involuntary admission between groups (28.3% vs. 33.3%, *OR* = 1.28, 95% CI [0.70–2.33], *p* = .430) for those where data were available. There was also no difference in the median number of bed days during the first year between the two groups (*Z* = −0.40, *p* = .692). These results were all consistent in the sub-groups of migrants classified according to continental region of birth (Table 3).

### Engagement with psychosocial interventions

The rates of attendance at CBT were similar between groups, with 52.6% (*n* = 41) of migrants and 51.8% (*n* = 146) of the Irish born cohorts attending CBT (*OR* = 1.03, 95% CI [0.63–1.71], *p* = .902). A total of 46.2% (*n* = 36) of caregivers for migrants attended the psychoeducation programme, compared to 39.7% (*n* = 112) of caregivers for the Irish born cohort (*OR* = 1.30, 95% CI [0.79–2.16], *p* = .307). These results were all consistent in the sub-groups of migrants classified according to continental region (Table 3).

## Discussion

### Summary of findings

The main findings of this study are that migrants have broadly similar outcomes to the native-born population across a wide range of outcomes, specifically remission rates and symptom severity, functioning, hospitalisation and engagement with psychosocial interventions. While this is encouraging that migrants are not disadvantaged in terms of experiencing poorer outcomes, further work needs to be done to improve the outcomes for both migrants and native-born groups.

### Comparison to previous literature

There are three main findings from this study that warrant further discussion and comparison to the previous literature, namely rates of remission, involuntary admission and engagement with psychosocial interventions. The high rates of symptomatic remission achieved in both groups is a striking finding, with over 77% of the total cohort being in remission of positive symptoms at the 1-year assessment point and 63.5% in remission of negative symptoms. These rates of remission are higher than that observed in a large meta-analysis, which found a pooled prevalence of remission of 58% (Lally et al., 2017), however it must be noted that we did not have data in relation to the duration of remission in this cohort. The findings of this study that there was no difference in the remission rates between migrants and the native-born population, replicates the results of Maguire et al. (2021) in Melbourne, Australia.

Although studies from Australia (Maguire et al., 2021), Canada (Rodrigues et al., 2019) and Italy (Tarsitani et al., 2022) have demonstrated that the migrant group are at higher risk for involuntary admission during follow-up periods after first presentation, our study did not replicate these findings, at least in the first year after presentation. There is also a strong body of evidence that migrants and ethnic minorities (Barnett et al., 2019; Terhune et al., 2022), have an increased risk of involuntary admission at the time of presentation. It is not yet known why migrants have an increased risk for involuntary treatment and the systematic review found that nearly half of studies offered no explanation for the observed variation in risk, and when explanations were provided, they were often untested or unproven (Barnett et al., 2019). It is likely that individual, health service and mental health legislation factors contribute to this variation in rates for involuntary admission. Therefore, comparison of the practices in different jurisdictions that have varying rates for involuntary admission in migrants, could reveal insights into possible explanations for the reasons for this practice. For example, it has been identified that the overall rate of involuntary admission is twice as high in England as compared to the Republic of Ireland and differences in definitions for a ‘mental disorder’, police involvement in the admission process, funding and access to psychiatric beds and perceptions of risk in the general public have been discussed as possible explanations (Conlan-Trant & Kelly, 2022).

Finally, the finding that the level of engagement with psychosocial interventions was no different to that of the native-born populations is reassuring. Overall, individuals with a FEP have higher engagement rates compared to standard adult mental health services (Bird et al., 2010). Interestingly, the absolute rates for attendance for CBT and caregiver psychoeducation were higher in the migrants’ groups, albeit, not statistically significant. While a number of clinical factors, such as a longer duration of untreated psychosis, poorer insight and more severe negative symptoms, are associated with poorer engagement with CBT (Alvarez-Jiménez et al., 2009; Fanning et al., 2012), this is the first study that we are aware of, that demonstrates that migrant status is not associated with poorer engagement or attendance for CBT. Although, in the UK, ethnicity has been shown to be associated with receipt of CBT, with individuals of African and Black-Caribbean ethnicity being less likely to receive CBT for psychosis (Morris et al., 2020). However, our study also highlights that only half of individuals with a FEP attended CBT and this supports findings from a recent UK study which found high disengagement rates (43%) from CBT in the total FEP population (Richardson et al., 2019). Therefore, the barriers to engaging with psychosocial interventions needs to be determined, in order to improve these engagement rates. There may be some barriers that are specific to migrants, such as unfamiliarity of how to access outpatient services, cultural relevance of interventions or not having proficiency in the English language, as there was no translator service available for the CBT treatment. These may be in addition to general barriers to engaging in psychosocial interventions, such as a perceived lack of need for the intervention, lack of transport, childcare or work obligations.

### Clinical implications

There are a number of important clinical implications that arise from this study. First, even though the outcomes were similar across migrants and the native-born population, further work on adapting interventions to specific populations could prove valuable (Degnan et al., 2018). Only half of the cohort engaged with CBT and different methods may be required to engage migrant populations in these psychological interventions. In the UK, it was demonstrated that CBT for psychosis and a family intervention could be adapted in a culturally acceptable manner for individuals of Black British, Black African, Black Caribbean ethnicity and Muslim Asian ethnicity and be effective in reducing symptoms severity (Edge et al., 2016; Rathod et al., 2013). Similarly, any barriers for the caregivers of migrants to attend and engage with the psychoeducation programme should be explored and addressed. Finally, the relatively high remission rates are encouraging but yet there is a significant proportion who continue to experience persistent psychotic symptoms 1 year after diagnosis. While there may be a number of reasons for these ongoing symptoms, such as non-compliance, it could be assumed that treatment resistance accounts for a proportion. Worryingly, there is evidence from a systematic review that clozapine is less likely to be prescribed to individuals from ethnic minorities when clinically indicated (Williams et al., 2020). While it could be commenced earlier if indicated, it is often after 1 year that clozapine is prescribed for individuals attending early intervention for psychosis services (Thien & O’Donoghue, 2019). Therefore studies with longer follow-up period are required to determine whether there are disparities in the prescribing of clozapine for migrants.

### Strengths and limitations

The main strength of this study is that a broad range of outcomes were measured using reliable and valid instruments and the original cohort consisted of all identified and treated cases of first episode psychosis within a defined catchment area and time-period. However, the findings need to be considered within the limitations of the study. First, over one-third of the original cohort were lost to follow-up and these individuals were more likely to be admitted at presentation, have more severe symptoms and less insight. All these factors can be associated with a poorer prognosis in FEP and therefore the cohort included in this follow-up study were biased towards those with more positive outcomes. Second, while this was a relatively large FEP cohort, the sub-groups of migrants were small when they were analysed according to their continent of birth and would have lacked statistical power to determine differences at this level. Related to this limitation, we only conducted analysis at the sub-group level of the continent of birth, as the small numbers did not permit examining the outcomes of migrants at the country of birth level. Furthermore, we were unable to differentiate individuals who were seeking asylum and those who were economic migrants and it is likely that these two groups would be quite distinct and could possibly have different outcomes.

## Conclusions

These findings demonstrate that migrants have broadly similar outcomes to the native-born populations, however there is still considerable scope for the outcomes for all individuals affected by psychotic disorders to be improved. In order to further maximise recovery rates for migrants, culturally adapted interventions could be developed and evaluated and implemented if found to be effective.
